# The construction of urban cultural and creative industries using deep learning and information management

**DOI:** 10.1016/j.heliyon.2024.e33787

**Published:** 2024-06-27

**Authors:** Zijian Zhao, Javier Garcia-Campayo, Jin Liang, Ruihui Pu, Hector Monzales Perez, Xi Xue, Luis Borao, Huaqiang Li, Argel Bondoc Masanda, Jing Chen, Lucila Matias Portugal, Jonathan Bulahan Aganan

**Affiliations:** a Yonsei University, Seoul, 03722, South Korea; b The Catholic University of Korea, Bucheon-si, 14662, South Korea; c University of Zaragoza, Zaragoza, 50009, Spain; d Lyceum of the Philippines University- Batangas, Batangas City, 4200, Philippines; e University of Chinese Academy of Social Sciences, Beijing, 102488, China; f University of San Miguel, Culiacán, 80020, Mexico; g The International Institute of Computing and Societal Education University, Wilmington, DE, 19899, USA; h Faculty of Economics, Srinakharinwirot University, Bangkok, 10110, Thailand; i Republic of the Philippines Professional Regulation Commission, Manila, 1008, Philippines; j Sichuan University of Media and Communications, Chengdu, 611745, China; k Fudan University, Shanghai, 200433, China; l National University Philippines, Baliwag, Bulacan, 3006, Philippines; m Xiamen Institute of Technology, Xiamen, 361021, China; n University of Santo Tomas, España Manila, 1008, Philippines

**Keywords:** Urban transformation, Deep learning, Long short-term memory, Cultural and creative industry, Development evaluation index, Psychological influencing factors

## Abstract

The ongoing transition within the Chinese economy assumes a pivotal role in shaping the landscape of the cultural and creative industries (CCI). Renowned for its environmentally sustainable attributes, coupled with high productivity, CCI has garnered considerable attention across diverse societal strata. This study endeavors to delineate the determinants influencing the developmental trajectory of CCI, with a focal point on City A as the primary subject of investigation, juxtaposed against Cities G, D, B, H, and X for comparative analysis, leveraging developmental data from the year 2021. Initially, the study elucidates the conceptual framework underpinning CCI and its intrinsic significance in facilitating urban metamorphosis. Subsequently, the positive impact of CCI through deep learning and information management technology is emphasized, and a cultural and creative recommendation model based on LSTM algorithm is constructed. Through performance evaluation, the recommendation accuracy for cultural and creative projects reaches 94.74 %. A robust developmental assessment model for CCI is then constructed via meticulous factor analysis of pertinent influencers. Employing factor analysis techniques, the study identifies two primary determinants exerting sway over CCI development: sustainable profitability factors and cultural influence factors. Noteworthy among the factors influencing CCI development within City A are fixed asset investment, cultural industry financing, the proliferation of university-based research institutions, and per capita cultural expenditure by residents. Of these, fixed asset investment, cultural industry financing, and the density of university research institutions prominently impinge upon sustainable profitability, with a discernible impact weight of 0.738 in the evaluative framework of CCI development, thus significantly shaping its trajectory. Moreover, consumer psychological factors, particularly market consumption patterns, are observed to exert a discernible influence on CCI evolution. This study augurs fresh insights into the realm of CCI development, infusing it with renewed vigor and vitality. Moreover, it underscores the inherent interdependence and positive correlation among the various research factors, offering novel perspectives and methodologies germane to the advancement of urban CCI.

## 1 Introduction

In recent years, the environmental ramifications stemming from economic activities such as energy consumption and industrial production have reached alarming levels. The conventional paradigm characterized by high input, elevated pollution, and low output has become untenable, necessitating urgent transformation. Concurrently, China's economic development trajectory has undergone a paradigm shift, with a growing emphasis on the tertiary sector. This shift underscores a concerted effort to enhance the overall efficiency of economic endeavors and foster a more harmonized economic development model [1]. Organizational behavior, as an interdisciplinary field, draws upon insights from management, psychology, sociology, anthropology, among others. It is dedicated to exploring the psychological and behavioral dynamics within organizational settings [2]. By shedding light on these dynamics, organizational behavior facilitates a deeper understanding of urban transformation and development processes.

Culture lies at the core of urban identity. As society progresses, culture has emerged as a primary driver of urban development, gaining increasing significance in shaping the trajectory of cities [3]. Cultural and Creative Industries (CCI), with their unique amalgamation across primary, secondary, and tertiary sectors, have emerged as pivotal drivers of global economic advancement and serve as catalysts for the modernization of nations. CCI fosters the growth of both upstream and downstream industries, facilitates industrial restructuring, and steers cities towards a transition from conventional to sustainable economies [4]. The development of CCI plays a pivotal and multifaceted role across various urban subsystems, including the economy, society, and culture, effectively propelling cities towards optimal growth and comprehensive transformation [5]. Research into the influencing factors of CCI development offers novel insights, bolstering industry competitiveness, accelerating urban power dynamics, and fostering the holistic development of economic and cultural life.

Against this backdrop, this study elucidates the role of CCI in urban transformation, grounded in a theoretical framework delineating the concept of CCI. It exemplifies the positive influence of deep learning and information management on CCI development and proposes influencing factors and specific metrics for CCI development. Furthermore, an evaluation model for CCI development is constructed based on these metrics. By focusing on City A as the primary research subject and comparing it with Cities G, D, B, H, and X, this study investigates the principal factors shaping CCI development through factor analysis.

This study contributes novel insights and methodologies in the examination of urban CCI construction. Compared to Lee et al.'s suggestion of conducting industry chain analysis on specific CCI before policy implementation, this study adopts the "relationship between production and reproduction" and "interactive consumption of the three-circle hypothesis" models to position CCI [6]. This aligns with the various factors that need to be considered in CCIdevelopment mentioned in this study, such as fixed asset investment and cultural industry funding. This study utilizes deep learning and information management techniques to design a cultural and creative recommendation model based on the LSTM algorithm. Based on this, a comprehensive evaluation model for the development of CCI is constructed, and an in-depth empirical analysis is conducted on the development of urban CCI. Primarily, leveraging deep learning and information management technology, this study assesses the significance of CCI in urban development. In contrast to conventional research methodologies, these advanced technologies possess superior data processing and predictive capabilities, facilitating a more precise delineation of trends and patterns in CCI development. Through the application of these methodologies, this study comprehensively scrutinizes the current state and challenges facing urban CCI, offering robust support for the formulation of effective developmental strategies. Furthermore, this study constructs an evaluation model for CCI development and evaluates urban CCI progression using empirical data. This model not only encompasses factors such as technological innovation, industry performance, and production parameters but also integrates dimensions such as market consumption and government support to holistically assess the level of CCIdevelopment. Compared to traditional evaluation approaches, the model proposed in this study is more scientifically grounded and objective, providing a precise reflection of the actual status of urban CCI. Lastly, this study conducts an exhaustive analysis of key factors influencing urban cultural and creative industry development, including technological innovation capabilities, market profitability, and governmental support. Through the examination of these factors, it unveils the underlying mechanisms driving urban CCI progression and offers actionable development strategies and recommendations for governmental entities and enterprises. Consequently, this study bears significant theoretical and practical implications for fostering the sustainable advancement of urban CCI. Through the application of deep learning and information management technology, it establishes a comprehensive evaluation model for cultural and creative industry development and conducts a thorough analysis of urban CCI evolution, presenting fresh insights and methodologies for related research domains. Amidst the ongoing urbanization process, this study holds pivotal significance in promoting the healthy growth of CCI and facilitating urban economic transformation and enhancement.

This study is structured into five distinct sections. The initial section of the introduction primarily delineates the research background and significance of the study while summarizing the research ideas and methodologies. Subsequently, the second section conducts a literature review, presenting scholars' research on CCI and elucidating the issues within this domain. The third section, method theory, elaborates on the concepts of organizational behavior and CCI, and formulates the development evaluation model of CCI. Following this, the fourth section outlines the experiment, with a focus on a specific city (referred to as "City A″), wherein a comparative analysis with several other regions is conducted, alongside an examination of City A's recent developmental trajectory utilizing factor analysis, culminating in the derivation of research outcomes. Finally, the fifth section encapsulates the conclusion, synthesizing the preceding discourse and identifying the limitations and shortcomings of the study. This study, centered on the determinants of CCI development, offers valuable insights for refining its developmental models and pathways.

## 2 Literature review

Despite CCI's status as a burgeoning industry, scholarly investigation has persisted. Liang and Wang, for instance, meticulously traced the evolution of CCI in China, discerned the impetuses driving its development, and assessed its ramifications on urban development and rejuvenation. Their inquiry encompassed a comparative analysis of the shared attributes between CCI in Chinese and Western cities, as well as the distinctive features of CCI within Chinese urban contexts. Furthermore, they proffered future research agendas aimed at elucidating the intricate urban development mechanisms influencing CCI and its multifaceted socioeconomic impact [7]. Their focus primarily centers on historical and contemporary analyses, lacking in-depth exploration concerning the concrete implementation of future development strategies and technological support. For instance, there remains a dearth of research on how to leverage deep learning and information management technologies to optimize the resource allocation and operational efficiency of CCI. Drawing insights from the strategic approaches adopted by CCI in the digital era, Benghozi et al. introduced two novel business models predicated on the reversal model and the traditional model of differentiated policies encountered by CCI enterprises. One model advocates a static yet adaptable strategy, while the other espouses a fluid framework characterized by proactive and agile tactics, aimed at adeptly navigating the challenges of the digital age [8]. The efficacy and operability of these models in specific applications still necessitate further validation and refinement, particularly within the context of big data and deep learning technologies. The translation of these strategies into actionable implementation plans remains a pressing issue awaiting resolution. Casey and O'Brien conducted an extensive investigation into the interrelationship between sociology and CCI, tracing its evolution from early empirical and theoretical antecedents to the formal establishment of CCI as a distinct research domain. Their study encompassed a comprehensive examination of the industry's coherence and utility, highlighting the reciprocal influence between sociology and CCI. Moreover, they deliberated on sociology's specific contributions to the broader study of CCI [9]. They tends to be more theoretical, with relatively less emphasis on the technical and managerial challenges encountered in the actual operation of CCI. Specifically, in terms of information management and technological applications, their research lacks concrete methodological guidance. Boix Domenech et al. undertook a comprehensive assessment of the overall impact of CCI on per capita income at national, regional, and urban levels across 78 developed and developing countries spanning five continents, as well as 275 European regions and 518 cities within Valencia, Europe. Employing data sourced from multiple databases and employing non-parametric local linear least squares methodologies, their analysis revealed CCI's significant role in enhancing the prosperity of both affluent and impoverished nations, albeit at the expense of exacerbating regional disparities and widening socioeconomic divides [10]. While they employ extensive data analysis to demonstrate the impact of CCI on income, there is still insufficient research on how to narrow regional disparities and enhance overall industrial benefits through technological means. Zhao conducted a meticulous review of literature spanning consumer psychology and the appreciation of cultural and creative artworks. Drawing from these sources, Zhao proposed a framework elucidating the emotional cognition of CCI within art museums under the influence of psychological factors. The findings underscored the profound impact of psychological determinants on consumers' emotional perception and consumption behavior within the realm of CCI [11]. His research primarily resided at the qualitative analysis level, lacking specific empirical studies on how to utilize deep learning techniques to quantify and predict the influence of these psychological factors. Alizadeh et al. proposed a hybrid model combining statistical and machine learning methods for short-term telecommunication network traffic prediction [12]. Although their applications differed, both employed deep learning technology to process and forecast data, focusing on enhancing the efficiency and effectiveness of specific systems through technological means. Pourvaziri et al. drove sustainable development in particular industries by identifying and addressing critical barriers, emphasizing the importance of policy support, market factors, technological applications, and knowledge training in the green procurement process [13]. Alizadeh et al. utilized a Convolutional Neural Network-Long Short-Term Memory (CNN-LSTM) hybrid algorithm as a classifier network, employing Firefly and Grey Wolf algorithms to optimize the hyperparameters of deep neural networks. They validated the effectiveness of the proposed intrusion detection strategy by comparing it with existing highly developed classifiers [14]. Zhan et al. integrated deep learning features with Criteria Importance Through Intercriteria Correlation (CRITIC) and Decision-Making Trial and Evaluation Laboratory (DMTEL) methods for assessing the development of low-carbon transportation systems, indicating the extensive application potential of deep learning technology in decision support systems across different fields [15]. Kamranfar et al. developed a new decision-making approach using DMTEL, Delphi technique, and Analytic Hierarchy Process to identify and evaluate factors influencing the development of green buildings [16].

Significant progress has been made in the field of the CCI. However, there still exists a notable research gap regarding how to leverage deep learning and information management technology to propel the development and optimization of the CCI. Hence, this study commences by elucidating the definitions of organizational behavior and CCI, underscoring the significance of CCI in urban transformation and development. It introduces specific metrics to gauge CCI development and consumer psychology, culminating in the establishment of a comprehensive evaluation model for CCI development. Focused on City A, the study delves into the determinants of CCI development utilizing factor analysis, facilitating horizontal comparisons across diverse regions and vertical assessments spanning multiple years within City A.

The novelty and contribution of this study lie in: integrating deep learning technology with information management to propose a novel CCI development evaluation model, thus addressing the existing technical application gap in current research. Through comparative studies across multiple cities, this study specifically identifies and analyzes the primary factors influencing CCI development, quantifying the impact of these factors and providing practical guidance for policymakers and businesses. Not only does this study focus on the economic benefits of CCIs, but it also emphasizes their cultural influence, thereby offering a more comprehensive evaluation framework. By utilizing empirical data from 2021 and employing quantitative analysis and empirical research, the effectiveness and reliability of the proposed model and methods are validated. Through these innovative contributions, this study not only enriches the theoretical foundation of CCI research but also provides practical and feasible technical methods for practice, injecting new vitality and momentum into the sustainable development of urban CCI.

## 3 Technical theory

### 3.1 The definition of CCI and its role in urban transformation and development

Numerous cities in China confront varying degrees of transformation and development, posing a significant challenge to the nation's social advancement. Urban transformation encompasses diverse modes, including ecological city models, smart city paradigms, cultural innovation frameworks, circular economy strategies, and more [17]. Despite their diversity, these modes share a common objective: diminishing the predominance of traditional industries in urban economic frameworks and transitioning towards the ascendancy of emerging industries [18].

Given the distinct economic developmental stages, the conceptualization of CCI varies across nations. In the United Kingdom, it is termed the creative industry, denoting a sector capable of generating wealth and employment through the cultivation and utilization of intellectual property rights stemming from individual creativity [19]. Conversely, the United States labels it the copyright industry, aligning with its emphasis on cultural copyright safeguarding [20]. Japan designates it a perceptual industry, esteeming CCI's services and cultural contributions [21]. Nonetheless, these conceptual extensions fundamentally converge, highlighting CCI's attributes of high technology integration, elevated added value, and environmentally sustainable practices [22]. In concurrence with China's CCI context, CCI is delineated as a substantial sector that innovates and enhances cultural assets leveraging advanced technology within a cultural milieu, yielding high-value products, and facilitating value exchange through market mechanisms.

The advancement of CCI can wield a positive and expansive impact on various facets of urban life encompassing the economy, society, culture, spatial dynamics, and environment, thereby fostering the sustainable evolution of comprehensive urban transformations [23]. Firstly, it fosters a shift in the urban economic growth paradigm, optimizing economic structures, and propelling urban economic metamorphosis. Secondly, it fosters the unfettered and holistic development of individuals, enhancing urban living standards, and propelling societal evolution. Thirdly, it fosters the perpetuation of urban heritage, augmenting urban cultural identity, and propelling cultural advancement. Fourthly, it catalyzes the transformation of central urban areas, spearheads the development of new urban domains, and refines urban spatial arrangements. Fifthly, it mitigates resource depletion, environmental pressures, and enhances the urban milieu.

### 3.2 The role of deep learning and information management in the development of CCI

Through extensive exploration of deep learning and information management, CCI have leveraged these technologies to foster the emergence of diverse downstream sectors. For instance, deep forgery technology employs deep learning-based face generation and manipulation techniques to synthesize authentic-looking forged faces, thereby catalyzing advancements in the entertainment and cultural industry [24]. Similarly, the utilization of CNN-enabled shot boundary detection and boundary frame target detection technology in the recording of intangible cultural heritage videos facilitates the propagation, preservation, and safeguarding of intangible cultural heritage [25]. CCI enterprises can construct information management systems tailored to specific requirements, reflecting the spatial dynamics of urban CCI development, showcasing urban cultural elements, and stimulating urban CCI growth [26]. Given the necessity for various facilities and structures in CCI development, accurately predicting the energy consumption of these buildings is imperative. Such scientific and effective energy consumption forecasting aids in timely equipment failure detection, precise energy dispatching, formulation of energy conservation management strategies, execution of energy conservation initiatives, and contributes to urban transformation and development endeavors [27].

Deep learning technologies, notably Generative Adversarial Networks (GANs), have found application in the creation of visual artworks, music, and written content. For instance, deep learning models can be trained to produce novel image style transfers or compose music tracks that embody specific emotions or styles, offering artists innovative tools to drive creativity in artistic expressions. By employing deep learning for consumer data analysis, companies in the CCI sector can gain deeper insights into their customer base, enabling them to offer personalized product recommendations and services tailored to individual preferences. For example, online video platforms utilize deep learning algorithms to analyze user behavioral data and predict movies or TV shows that align with users' preferences, thereby enhancing user satisfaction and platform engagement. By analyzing extensive datasets from social media, search engine queries, and online consumption patterns, deep learning models can identify prevailing cultural trends and anticipate future market demands. This intelligence is invaluable for CCI companies in devising strategic decisions, such as adjusting product portfolios and refining marketing approaches.

In the CCI domain, knowledge and information serve as fundamental assets. Through the establishment of efficient information management systems, enterprises can gather, store, and share critical information encompassing market research reports, customer feedback, and project documentation. This enhances team collaboration efficiency and expedites the product development lifecycle. With the pervasive adoption of digitalization, copyright protection has emerged as a significant concern in the CCI sector. Information management technologies like blockchain offer novel solutions for copyright registration, tracking, and transactions. Leveraging the immutable nature of blockchain, intellectual property can be safeguarded against unauthorized reproduction and distribution, while streamlining copyright transactions. Robust information management systems can amalgamate data from diverse sources, including sales figures, customer interactions, and social media engagements, empowering enterprises to gain profound insights into market dynamics and customer preferences. Informed by these analytical insights, companies can craft more targeted marketing strategies to bolster customer satisfaction and loyalty.

Currently, sensor technology is increasingly advanced, allowing for the collection of larger volumes of information data. This abundance of data serves as the foundation for prediction models based on deep learning algorithms. Deep learning network models encompass various architectures, including CNN, Recurrent Neural Networks (RNN), LSTM, and Stacked Autoencoders (SAE) [28]. As an illustration, the algorithmic workflow of deep learning is elucidated employing LSTM.

**Fig. 1:**
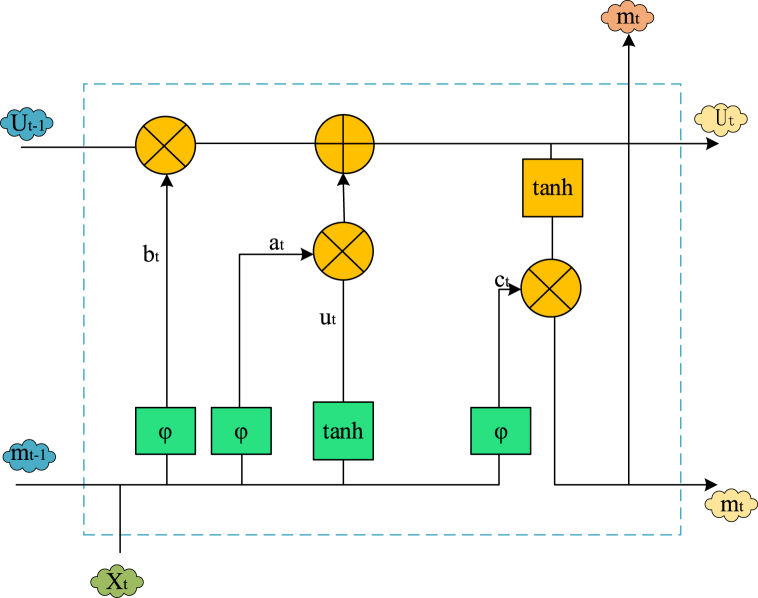
Structure of LSTM unit.

LSTM represents a specialized form of RNN. Within LSTM, the temporal node at the subsequent time step can retain historical information from preceding time steps over prolonged durations, enabling the processing of lengthy sequences of textual information. Fig. 1 illustrates the structural unit [29].

Fig. 1 illustrates that an LSTM neural unit comprises a memory unit and three gate units: input, forget, and output. Equations (1), (2), (3), (4), (5), (6) delineate each information update process of LSTM at time t [30].

(1) $$a_{t}=\phi \left[Q_{a}\left(m_{t-1},X_{t}\right)+w_{a}\right]$$

(2) $$b_{t}=\phi \left[Q_{b}\left(m_{t-1},X_{t}\right)+w_{b}\right]$$

(3) $$c_{t}=\phi \left[Q_{c}\left(m_{t-1},X_{t}\right)+w_{c}\right]$$

(4) $$U_{t}=\tan\;h\left[Q_{i}\left(m_{t-1},X_{t}\right)+w_{i}\right]$$

(5) $$U_{t}=b_{t}⨀u_{t-1}+a_{t}⨀U_{t}$$

(6) $$s_{t}=n_{t}⨀\tanh\;\left(u_{t}\right)$$

**Fig. 2:**
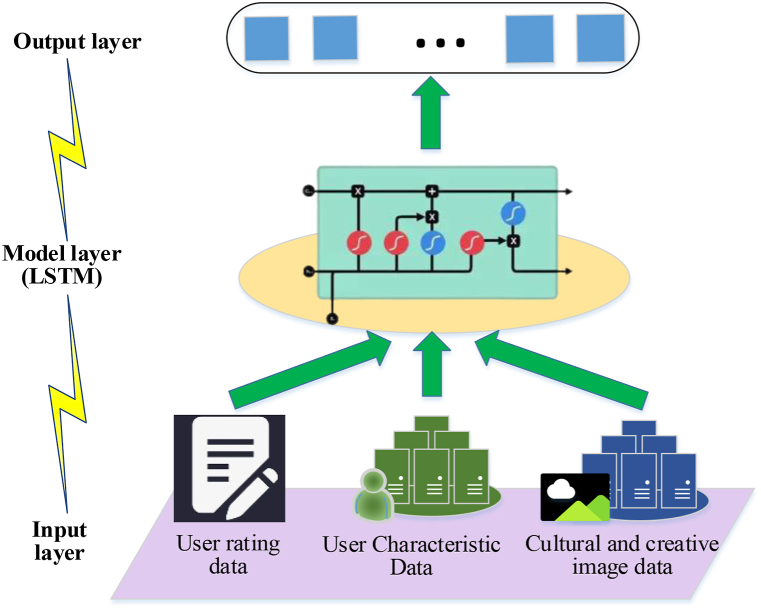
A cultural and creative recommendation model based on LSTM algorithm.

ϕ represents the sigmoid activation function, and ⨀ signifies the element-wise multiplication. $X_{t}$, $m_{t-1}$, and $u_{t-1}$ denote the input values at time *t*. $Q_{a}$, $Q_{b}$, $Q_{c}$, and $Q_{i}$ denote weight matrices, while $w_{a}$, $w_{b}$, $w_{c}$, and $w_{i}$ denote offset values. $a_{t}$ represents the input gate, responsible for determining whether the input information is stored in the current cell state or filtered out. $b_{t}$ denotes the forget gate, which filters out unimportant information from the previous moment. $c_{t}$ serves as the output gate, regulating the output of the current hidden layer information. $u_{t}$ signifies a memory unit, and $s_{t}$ pertains to a hidden state. The LSTM network employs a memory gate and a forget gate to update the information state of the storage unit at a specific time, effectively addressing the decline in perception ability of subsequent time nodes in RNN [31]. The integration of these deep learning algorithms can inject new impetus into the development of CCI and propel it toward a data-driven future. Fig. 2 presents the cultural and creative recommendation model framework based on LSTM algorithm constructed here.

As shown in Fig. 2, in this recommendation model, cultural and creative related data is first collected and preprocessed. The input data includes user ratings, user characteristics, and cultural and creative images. User rating data is used to capture the degree of user preference for creative products from different cultures. User characteristic data may include age, gender, geographic location, etc., to help the model understand the basic attributes of users. Cultural and creative image data helps understand the characteristics of products through visual features. The data preprocessing steps include normalizing user ratings, encoding user features, and performing image processing and feature extraction on image data. The processed data into the LSTM network, and the LSTM network can process sequential data, capture the trend of user behavior over time, and predict the potential interest of users in cultural and creative products.

During the model training process, network parameters are adjusted through optimization algorithms to minimize prediction errors. The specific hyperparameters of this model are as follows: training cycles (Epochs): 80, learning rate: 0.001, batch size: 128, activation function: ReLU, and optimizer: Adam.

Finally, the performance of the model is evaluated, accuracy is used as a metric to measure the effectiveness of the recommendation system, and the model is iteratively optimized based on feedback.

## 4 Evaluation model of cultural and creative industry development

**Fig. 3:**
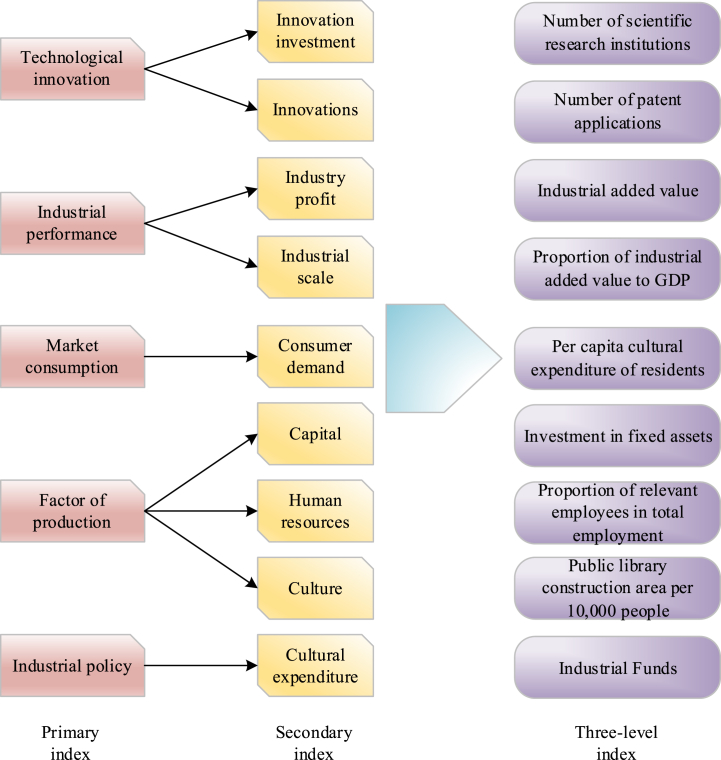
CCI development evaluation model.

The principal influencing factors of CCI development encompass technological innovation, industrial performance, production factors, market consumption, and industrial policy. Among these, scientific and technological innovation, industrial performance, production factors, and industrial policies constitute CCI development factors, while market consumption represents the consumer psychological aspect [32]. Building upon this foundation, a CCI development evaluation model is established, depicted in Fig. 3.

Fig. 3 illustrates that technological innovation constitutes a fundamental aspect of CCI development. The presence of scientific research institutions and the number of patent applications in colleges and universities serve as indicators reflecting the region's level of scientific advancement. Industry profitability and scale, quantified as CCI added value and the ratio of added value to gross domestic product (GDP), respectively, are crucial metrics for assessing industry performance. Factors of production, encompassing cultural, capital, and human factors, are also pivotal in CCI development and are measured by indicators such as the construction area of public libraries per 10,000 people, the number of fixed asset investments, and the proportion of related employees in total employment. Per capita cultural expenditure of residents serves as a reflection of the industrial consumption market's capacity, embodying consumer psychology and preferences toward CCI. The government's industrial policy, reflected in cultural industry funding, signifies governmental support for the sector.

Upon acquiring the requisite data from the statistical yearbook, the significant variation among indicators' dimensions necessitates standardization and conversion into comparable values within the same dimension. Equation (7) outlines the calculation method for achieving this standardization [33]:

(7) $$s_{mn}^{*}=\frac{s_{mn}-s_{n}}{l_{n}}$$

In Equation (7), $s_{mn}^{*}$ represents the standardized value, $s_{mn}$ denotes the original data, $s_{n}$ signifies the average of the *n*th indicator, and $l_{n}$ represents the standard deviation of the *n*th indicator. Subsequently, eigenvalues, variance contribution rates, and cumulative variance contribution rates are computed for each index to ascertain the principal factor. The weight of the principal factor is then determined based on the variance contribution rate and the cumulative variance contribution rate, as elucidated in Equation (8).

(8) $$w_{i}=\frac{V_{i}}{C_{i}}$$

In Equation (8), $w_{i}$ denotes the weight of the *i*i-th principal factor, $V_{i}$ signifies the variance contribution rate of the factor, and $C_{i}$ represents the cumulative variance contribution rate of the factor. Leveraging the weights of the principal factors, the score of each variable on the principal factor can be derived. Assuming the original *n* variables are denoted as z_1_, z_2_, z_3_, …, and z_n_, while the principal factors extracted through factor analysis are denoted as F1, F2, F2, …, and Fi, the methodology for calculating the score of each variable is articulated through Equations (9), (10), (11) [34].

(9) $$Z_{1}=\alpha_{11}F1+\alpha_{12}F2+\cdots +\alpha_{1i}Fi+\alpha_{1}\theta_{1}$$

(10) $$Z_{2}=\alpha_{21}F1+\alpha_{22}F2+\cdots +\alpha_{2i}Fi+\alpha_{2}\theta_{2}$$

(11) $$Z_{n}=\alpha_{n1}F1+\alpha_{n2}F2+\cdots +\alpha_{ni}Fi+\alpha_{n}\theta_{n}$$

In Equations (9), (10), (11), ***α*** represents the factor loading, signifying the relationship between the extracted principal factor and the original variable. A higher value of ***α*** denotes a greater explanatory power of the principal factor concerning the original variable. Moreover, ***θ*** denotes a distinct factor that cannot be explicated by the principal factor.

## 5 Experimental design and performance evaluation

### 5.1 Datasets collection, experimental environment and parameters setting

A comprehensive study encompasses six cities, comprising City G and City D characterized by robust economic development, followed by City A, City B, and City H exhibiting moderate economic progress, and finally, City X with comparatively underdeveloped economic status. Notably, City A serves as the primary subject of investigation, while the remaining five cities serve as comparative references. To ensure methodological rigor, data are sourced from regional statistics released by the National Bureau of Statistics of China, alongside information from the statistical yearbook of Chinese culture and related industries [35]. Table 1 presents the descriptive data pertaining to the six cities for the year 2021 [36].

**Table 1:** Statistical table of various indicators in 2021.

Cities	City A	City G	City B	City D	City H	City X
Z1 Number of scientific research institutions in universities	144	152	145	139	140	127
Z2 patent applications (thousands)	193.31	200.44	195.20	192.45	187.87	166.79
Z3 CCI added value (100 million yuan)	2480	2703	2612	2479	2475	2004
Z4 added value as a percentage of GDP (%)	00685	0.0751	0.0672	0.0681	0.0671	0.0553
Z5 Public library building area per 10,000 people (m^2^/10,000 people)	73	84	75	72	71	62
Z6 Fixed asset investment (100 million yuan)	1673.67	2202.43	1788.91	1669.62	1656.45	1403.84
Z7 Proportion of related employees in total employment (%)	0.86	0.95	0.77	0.85	0.84	0.67
Z8 Per capita cultural expenditure of residents (yuan)	1656.1	2000.3	1800.4	1654.4	1755.2	1533.8
Z9 cultural industry funding (100 million yuan)	29.97	40.01	37.86	30.88	35.45	20.51

Table 1 delineates the comparative analysis of various indicators among the six cities in the year 2021. It reveals that City A boasts a prominent position across several metrics, including the number of scientific research institutions in universities, patent applications, added value of CCI, proportion of CCI added value in GDP, building area of public libraries per 10,000 people, investment in fixed assets, and the proportion of relevant employees in total employment, ranking within the top three compared to other cities. However, City A still exhibits a disparity with City G, which demonstrates superior performance across these indicators, thereby positioning City G as the foremost in terms of development. Notably, City A records a per capita cultural expenditure of 1656.1 yuan, placing it fourth among the six cities, indicating relatively subdued market demand. Furthermore, the expenditure on the cultural industry in City A stands at 2.997 billion yuan, ranking fifth among the six cities, suggesting comparatively lower government support.

The study employs a 64-bit Windows 10 operating system, featuring an Intel Xeon (R) CPU E5-2650 V4 @ 2.20Hz × 15 as the central processing unit (CPU), 126 GB of running memory, a GTX 1080 graphics card, and a 5.2 TB storage hard disk. Data processing is conducted using the SPSS software without necessitating any parameter settings.

### 5.2 Performance analysis of recommendation systems under different algorithms

**Fig. 4:**
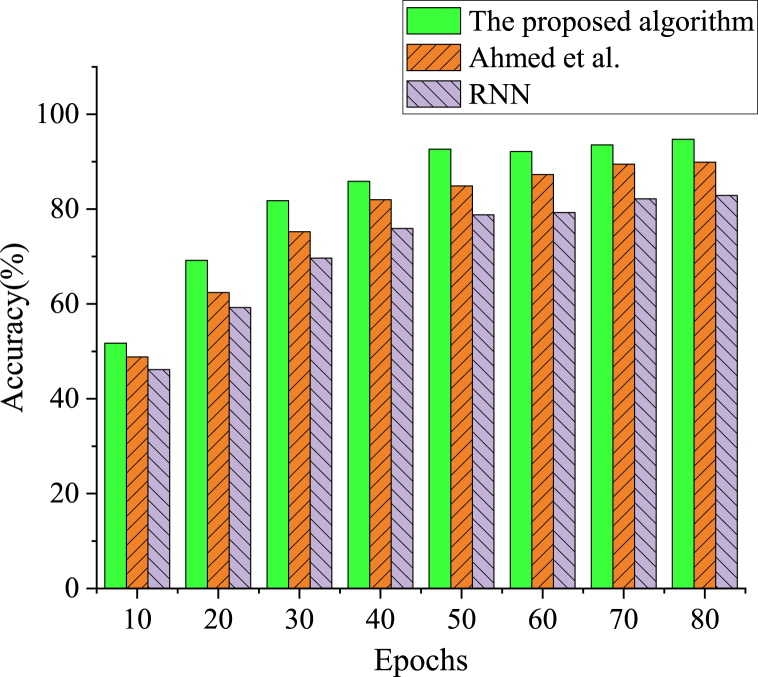
Accuracy results with cycle variation for each algorithm.

Compare the system model constructed here with RNN and the model algorithm proposed by Ahmed et al. (2021) [37] in related fields, and analyze it using the Accuracy index, as shown in Fig. 4.

As shown in Fig. 4, the accuracy of cultural and creative project recommendation is compared and analyzed between the proposed algorithm and other algorithms based on the Accuracy index. The accuracy of the proposed algorithm reaches 94.74 %, which is at least 4.82 % higher than the model algorithms proposed by other scholars. The recommendation prediction accuracy of the cultural and creative recommendation model based on LSTM algorithm constructed here is better.

### 5.3 Performance evaluation

Subsequent to standardizing the original data of the six cities, the correlation coefficient matrix of each variable, denoted as Z1-Z9, is computed. The outcomes of this analysis are presented in Table 2.

**Table 2:** Correlation coefficient matrix.

	Z1	Z2	Z3	Z4	Z5	Z6	Z7	Z8	Z9
Z1	1	0.861	0.873	0.382	0.095	0.788	0.439	0.124	0.826
Z2	0.861	1	0.940	0.567	0.168	0.614	0.860	0.333	0.862
Z3	0.873	0.940	1	0.726	0.164	0.686	0.745	0.471	0.927
Z4	0.382	0.567	0.726	1	0.211	0.255	0.813	−0.058	0.566
Z5	0.095	0.168	0.164	0.211	1	−0.251	−0.014	0.476	−0.155
Z6	0.788	0.614	0.686	0.255	−0.251	1	0.393	−0.591	0.805
Z7	0.439	0.860	0.745	0.813	−0.014	0.393	1	0.572	0.737
Z8	0.124	0.333	0.471	−0.058	0.476	−0.059	0.572	1	0.279
Z9	0.826	0.862	0.927	0.566	−0.155	0.805	0.737	0.279	1

Table 2 illustrates the correlation among the variables, revealing that the majority exhibit significant correlations, with most absolute values surpassing 0.3. This observation suggests a robust interrelationship among the variables, thereby affirming the judiciousness of variable selection for subsequent factor analysis.

**Fig. 5:**
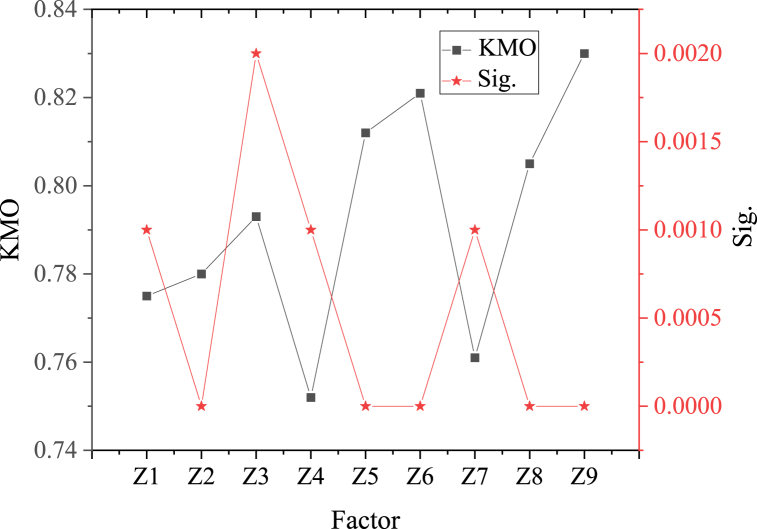
Validity test results for each factor.

Firstly, to demonstrate the reliability of each factor, KMO and Bartlett's sphericity tests are conducted, as shown in Fig. 5.

Fig. 5 suggests that, in the validity test of each factor, the KMO values of each factor are not less than 0.750, and the Sig. values are all less than 0.050. This indicates that the factors selected here have obvious internal consistency and stability, and high credibility. This can be used as a basis for further research.

**Fig. 6:**
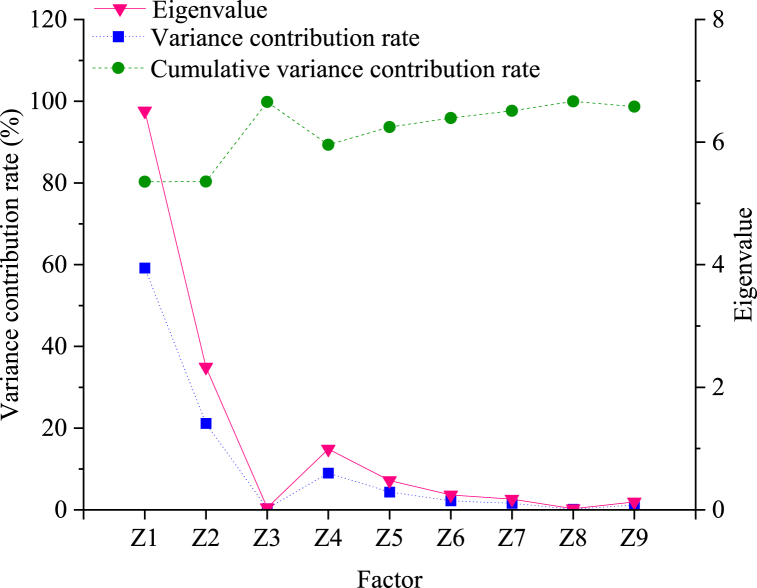
Eigenvalue, variance contribution rate and cumulative variance contribution rate of each factor.

Following this, the eigenvalues, variance contribution rates, and cumulative variance contribution rates for each factor are computed. The outcomes of these calculations are visually represented in Fig. 6.

Fig. 6 depicts the eigenvalues of the first and second factors, measuring 6.508 and 2.329, respectively. Both values exceed 1, thus warranting extraction as primary factors. The variance contribution rates associated with these main factors stand at 59.17 % and 21.16 %, with cumulative variance contribution rates of 80.29 % and 80.35 %, respectively. This signifies that the combined interpretation of the main factors towards the original variables reaches 80.35 %, indicating a substantial encapsulation of information from the majority of variables.

**Fig. 7:**
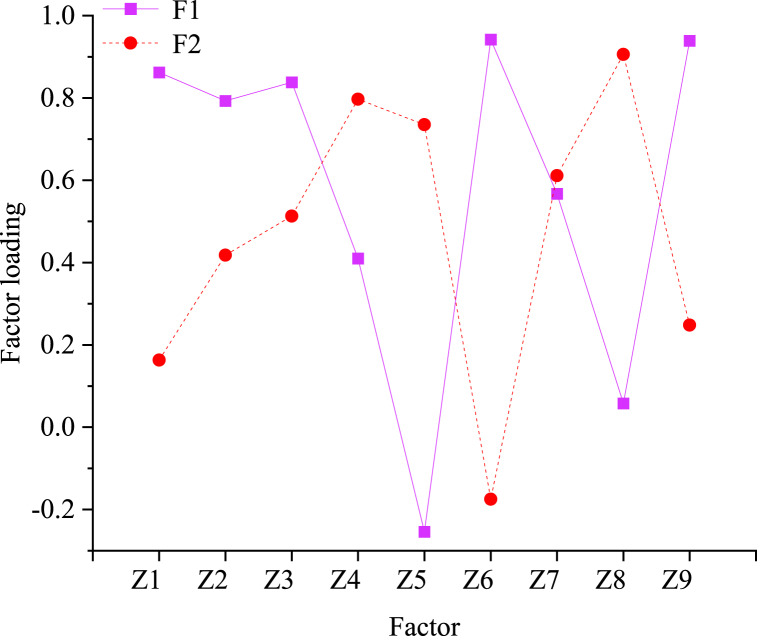
Factor loadings after rotation.

To enhance the significance of the distribution of the original variables on the main factor, the maximum variance method is employed to rotate and transform the loading matrix. The resulting rotated factor loading is presented in Fig. 7.

Fig. 7 illustrates that indicators Z1, Z2, Z3, Z6, and Z9 exhibit higher loadings on the main factor F1, representing the number of scientific research institutions in universities, the number of patent applications, the added value of CCI, the number of investments in fixed assets, and the expenditure of cultural industries, respectively. Scientific and technological innovation stands as the cornerstone of CCI, while factors such as production, industrial policies, and industrial performance collectively determine the market profitability of CCI. Consequently, F1 effectively consolidates the primary information encompassing these factors, thereby earning the designation of the sustainable profitability factor. Conversely, indicators Z4, Z5, Z7, and Z8 demonstrate higher loadings on the main factor F2, notably comprising the proportion of added value in GDP, the construction area of public libraries per 10,000 people, the proportion of related employees in total employment, and the per capita cultural expenditure of residents. Notably, per capita cultural expenditure of residents exhibits the highest factor loading of 0.906, underscoring its significant influence on F2. Consumer demand serves as a pivotal indicator of CCI's impact on economic and cultural realms, thus F2 is termed the cultural influence factor.

Based on the variance contribution rate and cumulative variance contribution rate of F1 and F2, their respective weights are determined. The sustainable profitability factor F1 bears a weight of 0.738, whereas the cultural influence factor F2 carries a weight of 0.262. This analysis indicates that the sustainable profitability factor exerts a more pronounced influence on the development of CCI in City A.

Subsequently, scores for each city under the main factors are computed using factor loadings and standardized variables. Denoted as x1-x9, these standardized variables are represented by the expressions: *F*1 = 0.147*x*1+0.113*x*2+0.111*x*3+0.005*x*4−0.109*x*5+0.196*x*6+0.052*x*7−0.073*x*8+0.154*x*9*,* and *F*2 = −0.016*x*1+0.092*x*2+0.125*x*3+0.276*x*4+0.273*x*5−0.156*x*6+0.187*x*7+0.35*x*8−0.012*x*9. Furthermore, the overall evaluation function for each city is derived, with City A exemplified by the expression: *Y* = 0.738*F*1+0.262*F*2*Y* = 0.738*F*1+0.262*F*2. Employing the same methodology, the overall evaluation functions for City B, City D, City G, City H, and City X are determined.

**Fig. 8:**
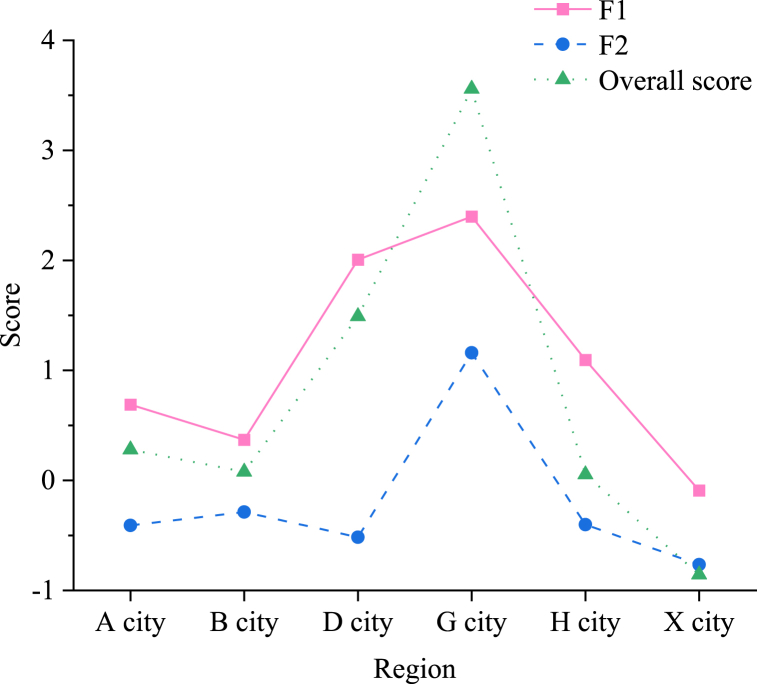
Main factor scores and comprehensive scores in 6 regions.

Subsequently, the normalized data is integrated into the function to obtain the overall scores of City A and the remaining five regions, as depicted in Fig. 8.

Fig. 8 illustrates the overall score of CCI in City A for the year 2021, which stands at 0.281, ranking third among the six regions. This score markedly diverges from the score of City G (3.558), which leads the first echelon, indicating that the development of CCI in City A still lags behind the top tier in China. However, City A's relative strength ranks high, signifying a robust development of CCI relative to other regions, albeit with room for further enhancement. The score for the sustainable profitability factor is 0.689, indicating a relatively strong capacity for continuous innovation and market profitability within CCI in City A. Conversely, the score for the cultural influence ability factor is −0.408, suggesting a current weakness in City A's cultural influence capacity.

### 5.4 Factors influencing sustained profitability and cultural influence ability

**Fig. 9:**
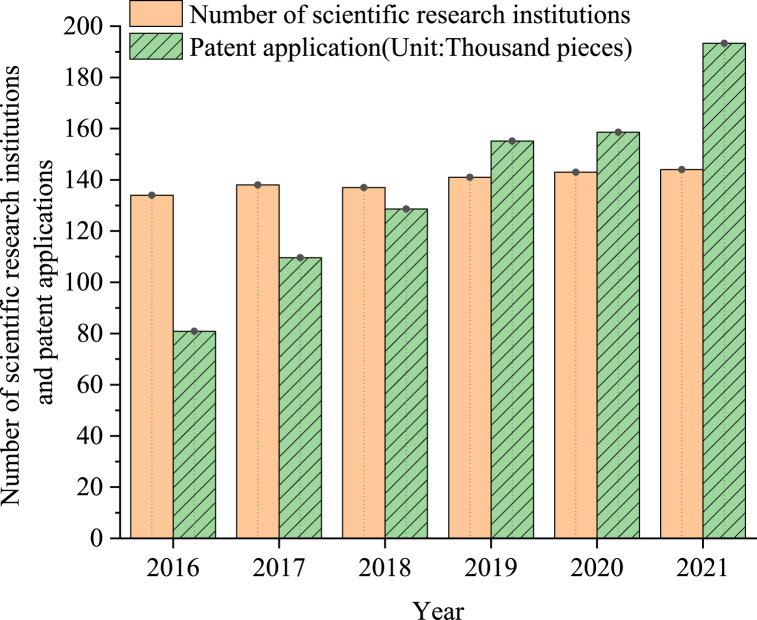
The number of scientific research institutions and patent applications in city A from 2016 to 2021.

The sustainable profitability factor primarily assesses the capacity for continuous innovation and market profitability, while cultural influence ability pertains to CCI's capacity to reflect external impacts. The number of scientific research institutions and patent applications in City A's universities serve as indicators of scientific and technological innovation capacity. Data on the number of scientific research institutions and patent applications in City A from 2016 to 2021 are sourced from the statistical yearbook, as depicted in Fig. 9.

Fig. 9 illustrates the steady improvement in the scientific research capabilities and technological level of City A in recent years. Particularly noteworthy is the significant increase in patent applications, surpassing 100,000 in 2017 and exceeding the 1.5 million mark in 2019. The upward trend in patent applications continues unabated, indicating ongoing advancements. Although the number of scientific research institutions in universities appears to exhibit minimal fluctuation, with a slight decrease observed in 2018 compared to 2017, the overall trend shows a consistent increase each year. As of 2021, the number of scientific research institutions in City A's universities surpasses 140, with these institutions serving as crucial hubs for nurturing creative talents. In essence, City A boasts a commendable talent strategy and a conducive scientific research environment, laying a solid foundation for the development of CCI and consistently infusing fresh vitality.

**Fig. 10:**
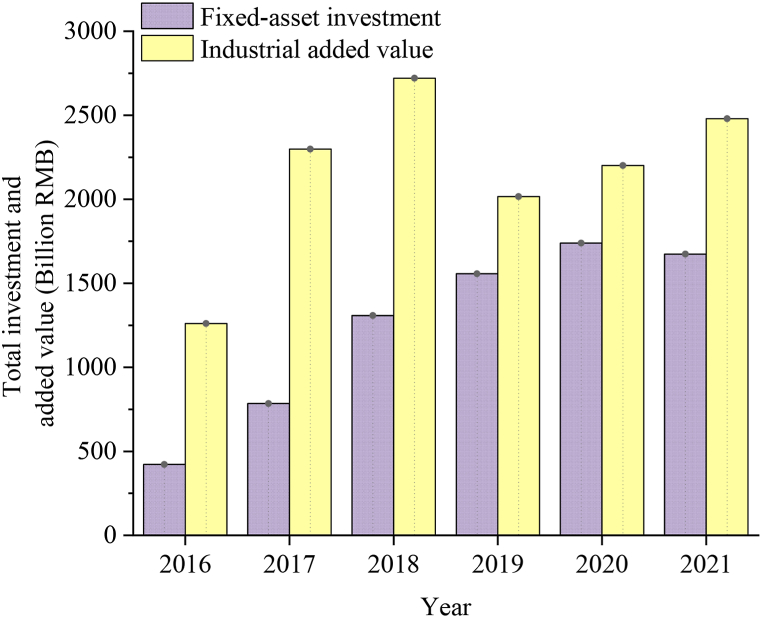
CCI fixed asset investment and industrial added value in City A from 2016 to 2021.

Market profitability, indicative of CCI's fixed asset investment and added value, is depicted in Fig. 10.

Fig. 10 suggests that the investment in fixed assets within CCI in City A has exhibited a steady increase over the past six years, experiencing only a minor decline in 2021, which is 0.038 % lower than that in 2019. However, the developmental trajectory of industrial added value over the same period is less favorable. Notably, there is a significant decline in 2019, plummeting by 25.9 % compared to 2018. This downturn indicates that the development of CCI in City A encounters a bottleneck, potentially stemming from issues related to resource allocation and utilization. Economic factors serve as the foundation for the sustainable development of CCI, wherein higher investment in fixed assets typically correlates with greater industrial added value.

**Fig. 11:**
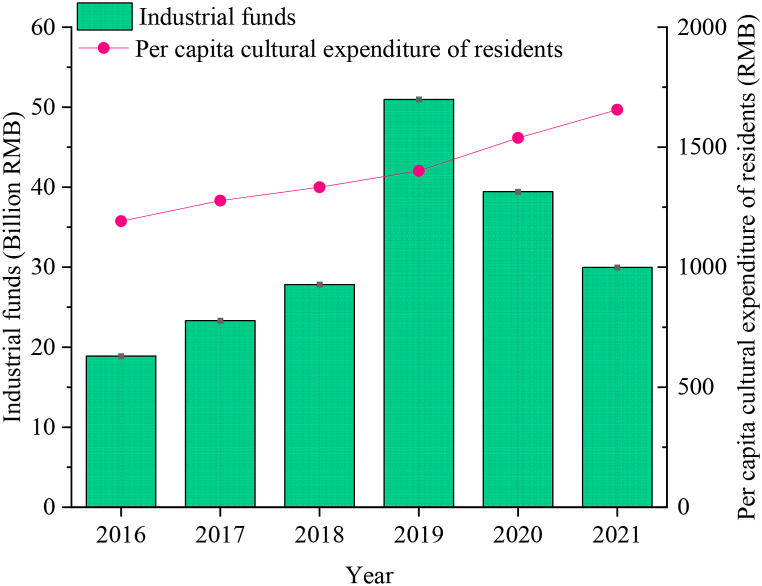
2016–2021 CCI industry expenditure and per capita cultural expenditure of residents in City A.

Government support manifests in the allocation of funds for the cultural industry by governmental entities. The per capita cultural expenditure of residents, which holds significant sway over cultural influence ability, serves as a barometer of market demand within the CCI sector. The statistical outcomes pertaining to these two factors are presented in Fig. 11.

Fig. 11 depicts the notable increase in appropriation for the cultural industry in City A in recent years, particularly evident post-2017 where the growth rate became more pronounced. In 2019, the appropriation surpassed 5 billion yuan. This sustained support has gradually steered the CCIin City A onto a positive trajectory, with the market-oriented mechanism progressively maturing. Concurrently, the per capita cultural expenditure of residents has exhibited an upward trend over the years. This trend indicates that the CCI market in City A is continually expanding, thereby providing a solid foundation for the long-term development of CCI.

### 5.5 Discussion

The outcomes reveal that the CCI development evaluation model offers insights into industry growth strategies. Furthermore, the findings are juxtaposed with existing research. Imperiale et al. employed a two-step hybrid approach to scrutinize regional specialization alongside CCI's entrepreneurial structure and business sustainability, identifying CCI's financing capabilities, local policies, and funding requisites as primary determinants of its development [38]. This aligns with the present study's results, highlighting the influential role of policies and production factors in CCI development. Similarly, Wu and Lin, adopting an urban control perspective, integrated entropy weight and grey relational degree methodologies to assess the evaluation index system, emphasizing the positive influence of government policies and resource allocation implementation on CCI development [39]. This concurs with our findings, underlining the impact of government policies on CCI development. Conversely, Bertoni et al., focusing on European cities' cultural and creative vitality, creative economy, and environmental aspects, proposed the application of urban spatial context, highlighting its close association with CCI comprehension and urban development [40]. While divergent in their spatial dimension approach, it aligns with our study's outcomes, emphasizing the influence of policy and technological innovation on CCI development. However, this study also unveils the impact of market consumption and industry performance, aspects not addressed in prior studies. Hence, the formulated CCI development evaluation model stands poised to optimize CCI development and foster robust growth initiatives.

### 5.6 Strategies and recommendations

In the future development of the CCI in City A, the following recommendations should be considered.

(1) Strengthening the Integration of Technology and Culture: City A should continue to promote the integration of advanced technologies such as deep learning and information management with the CCI to foster industry innovation and enhance product competitiveness.
(2) Optimizing Resource Allocation: It is recommended that policymakers in City A optimize fixed asset investment and allocate funds to the cultural industry to support key areas of the CCI and start-up enterprises, thus promoting balanced industrial development.
(3) Enhancing Residents' Cultural Consumption: By enhancing education and marketing activities, City A should increase residents' awareness of and willingness to consume cultural products, expand the cultural consumption market, and enhance the market's self-growth capability.
(4) Strengthening Talent Cultivation and Recruitment: City A needs to prioritize the cultivation and recruitment of cultural and creative talents by establishing talent cultivation bases through cooperation with higher education institutions, attracting and retaining highly skilled personnel.
(5) Promoting Regional Synergistic Development: City A should establish closer cooperation with surrounding cities, share resources, and form a synergistic effect in the regional CCI.
(6) Strengthening Copyright Protection: City A should intensify efforts to protect intellectual property rights, providing a secure environment for the creation and transaction of cultural and creative works, thus stimulating innovation vitality.
(7) Promoting Green and Sustainable Development: In the process of CCI development, emphasis should be placed on ecological protection and resource conservation, promoting green production and consumption patterns to achieve a win-win situation for economic development and environmental protection.
(8) Establishing a Dynamic Monitoring and Evaluation Mechanism: It is recommended to establish a dynamic monitoring system to regularly evaluate the development status of the CCI and promptly adjust and optimize policy measures.

By implementing the above recommendations, City A can not only promote the sustainable and healthy development of the CCI but also enhance the overall competitiveness of the city, thus achieving comprehensive socioeconomic progress.

## 6 Conclusion

To explore the influencing factors of CCI development in urban transformation, this study commences with an elucidation of the CCI concept and underscores its pivotal role in urban transformation. Thus, a cultural and creative recommendation model based on LSTM algorithm is constructed, and a comprehensive evaluation model for the development of CCI is constructed on this basis. The catalytic effect of deep learning and information management technology on CCI development is substantiated through illustrative examples. Subsequently, an evaluation model for CCI development is formulated, incorporating factors such as scientific and technological innovation, industrial performance, production factors, market consumption (consumer psychology), and industrial policy. Utilizing factor analysis, the following conclusions are derived: (1) The paramount factors influencing CCI development are identified as sustainable profitability and cultural influence. Notably, the influence weight of sustainable profitability is determined to be 0.738, underscoring its significant impact on CCI development. (2) Consumer psychology, epitomized by market consumption, exhibits a discernible impact on CCI development, with both factors demonstrating a positive correlation and interdependency. The advancement of CCI stimulates residents' cultural expenditure, reciprocally fostering the development of CCI.

While this study has delved into the development of urban CCI and proposed a comprehensive evaluation model, it still has some limitations. Firstly, the study only selects specific cities in China as research subjects, thus limiting the universality of the research results, which may not necessarily apply to other countries or regions. Secondly, the data used in this study primarily comes from official statistical yearbooks and data reports, which may suffer from incompleteness or inaccuracy, thereby affecting the reliability of the research results. Additionally, this study only considers some of the factors influencing the development of urban CCI, failing to conduct a comprehensive analysis of all potential factors. Therefore, future studies could consider using more diverse data sources and more comprehensive factor analysis methods to obtain more accurate and comprehensive research conclusions.

Furthermore, this study primarily focuses on constructing a development evaluation model for urban CCI using deep learning and information management technologies. However, future experiments could further explore the application of integrated decision support tools in CCIdevelopment, such as hybrid methods combining CRITIC-DEMATEL with deep learning features, as well as mixed decision-making methods using DEMATEL and ANP solutions. The CRITIC-DEMATEL method combines two techniques: CRITIC is used to determine the weights of various indicators, while DEMATEL is used to analyze the causal relationships between indicators. This method is suitable for identifying and addressing complex problems and obstacles in CCI development. When combined with deep learning algorithms, this hybrid method can not only reveal the constraining factors of CCI development from multiple dimensions but also utilize the data mining capabilities of deep learning to automatically learn and predict industry development trends and outcomes. Similarly, the mixed decision-making method combining DEMATEL with ANP can help us understand the challenges in CCI development. ANP, as a widely used network structuring technology for decision-making problems, can be used to address dependencies and feedback issues among various elements within the CCI industry. Through this methodological analysis, the dynamic complexity of CCI development can be more intuitively mapped out, supplemented by deep learning features, to provide effective strategic recommendations for the sustainable development of the CCI industry. Based on the above analysis, future study will focus on the application and promotion of these composite technologies, as well as how to effectively integrate them with deep learning features to provide more powerful decision support tools for the transformation and development of urban CCI.

## Data availability statement

Data will be made available on request.

## CRediT authorship contribution statement

**Zijian Zhao:** Writing – original draft, Formal analysis, Data curation, Conceptualization. **Javier Garcia-Campayo:** Software, Resources, Methodology, Investigation. **Jin Liang:** Writing – review & editing, Visualization, Validation, Supervision. **Ruihui Pu:** Writing – review & editing, Visualization, Validation, Supervision. **Hector Monzales Perez:** Resources, Project administration, Formal analysis. **Xi Xue:** Software, Resources, Project administration. **Luis Borao:** Methodology, Formal analysis, Data curation. **Huaqiang Li:** Visualization, Validation, Software. **Argel Bondoc Masanda:** Supervision, Project administration, Investigation. **Jing Chen:** Supervision, Software, Project administration. **Lucila Matias Portugal:** Methodology, Formal analysis, Data curation, Conceptualization. **Jonathan Bulahan Aganan:** Methodology, Funding acquisition.

## Declaration of competing interest

The authors declare that they have no known competing financial interests or personal relationships that could have appeared to influence the work reported in this paper.
